# Calcium Silicate-Based Root Canal Sealers: A Narrative Review and Clinical Perspectives

**DOI:** 10.3390/ma14143965

**Published:** 2021-07-15

**Authors:** Germain Sfeir, Carla Zogheib, Shanon Patel, Thomas Giraud, Venkateshbabu Nagendrababu, Frédéric Bukiet

**Affiliations:** 1 Department of Endodontics, Faculty of Dental Medicine, Saint Joseph University of Beirut, Beirut 17-5208, Lebanon; germain.sfeir@usj.edu.lb (G.S.); carla.zogheibmoubarak@usj.edu.lb (C.Z.); 2 King’s College London Dental Institute, Guy’s Tower, Guy’s Hospital, St. Thomas’ Street, London SE1 9RT, UK; shanonpatel@gmail.com; 3 Assistance Publique des Hôpitaux de Marseille, 13005 France; Aix Marseille Univ, CNRS, ISM, Inst Movement Sci, 13288 Marseille, France; thomas.giraud@univ-amu.fr; 4 Department of Preventive and Restorative Dentistry, College of Dental Medicine, University of Sharjah, Sharjah 27272, United Arab Emirates; hivenkateshbabu@yahoo.com

**Keywords:** calcium silicate-based root canal sealer, hydraulic root canal sealer, root canal obturation, root canal treatment

## Abstract

Over the last two decades, calcium silicate-based materials have grown in popularity. As root canal sealers, these formulations have been extensively investigated and compared with conventional sealers, such as zinc oxide–eugenol and epoxy resin-based sealers, in in vitro studies that showed their promising properties, especially their biocompatibility, antimicrobial properties, and certain bioactivity. However, the consequence of their higher solubility is a matter of debate and still needs to be clarified, because it may affect their long-term sealing ability. Unlike conventional sealers, those sealers are hydraulic, and their setting is conditioned by the presence of humidity. Current evidence reveals that the properties of calcium silicate-based sealers vary depending on their formulation. To date, only a few short-term investigations addressed the clinical outcome of calcium silicate-based root canal sealers. Their use has been showed to be mainly based on practitioners’ clinical habits rather than manufacturers’ recommendations or available evidence. However, their particular behavior implies modifications of the clinical protocol used for conventional sealers. This narrative review aimed to discuss the properties of calcium silicate-based sealers and their clinical implications, and to propose rational indications for these sealers based on the current knowledge.

## 1. Introduction

Despite numerous technological leaps, the purpose of root canal treatment is still prevention and healing of apical periodontitis by achieving proper disinfection and three-dimensional filling of the root canal space [1]. Root canal filling prevents diffusion of microorganisms and their byproducts and has been subject to various modifications from the use of solid material to gutta-percha cones in association with root canal sealers [2]. Various types of root canal sealers have been developed, such as zinc oxide–eugenol, epoxy resin, glass ionomer, and silicone-based sealers [3]. In the last decade, calcium silicate-based sealers (CSBS), often called “bioceramic” sealers, have been released and extensively investigated by comparing their properties to those of zinc oxide–eugenol-based and epoxy resin-based sealers [4,5]. Many formulations are available on the market. Unlike conventional root canal sealers, CSBS are hydraulic and hygroscopic with a particular setting process [6]. CSBS exhibit several interesting properties, especially biocompatibility, antimicrobial properties, and bioactivity [7,8,9,10,11,12]. Nevertheless, the dimensional stability of CSBS showed contradicting results among studies; while some studies showed no shrinkage upon setting, other demonstrated a slight expansion [3,4]. Mineral layer formation during setting induces a chemical bond with dentin walls in biological environment, which contributes to their sealing ability [4,5,6].

To date, if laboratory studies showed favorable results regarding CSBS’ physico-chemical and biological properties [13,14,15,16,17,18], only a few short-term investigations addressing the clinical outcome of CSBS have been published [19,20,21]. Moreover, a recent survey demonstrated that the methods of using CSBS in clinical practice were variable and based on practitioners’ habits rather than manufacturers’ recommendations or available evidence on these sealers [22]. This highlights the possible inappropriate use of CSBS, which may negatively impact the obturation, and thus the outcome of the root canal treatment. Moreover, this exposes a knowledge gap between the fundamental research on CSBS and their clinical application, justifying the need to better connect these two aspects. The number of CSBS formulations is strongly increasing over time, so it is of prime importance to better understand their specificities and their clinical perspectives.

Hence, the current review aimed to discuss the properties of CSBS and their clinical implications, and to propose rational indications based on the current knowledge and CSBS specificities.

### 1.1. Literature Search Methodology

Two independent reviewers (G.S., C.Z.) performed a comprehensive literature search to identify related studies in PubMed, Scopus, Web of Science, and Cochrane Library databases, between 1 January 2010 and 15 May 2021. The following search strategy was used to find relevant studies: (bioceramic sealer OR bioceramic root canal sealer) OR (hydraulic sealer OR hydraulic root canal sealer) OR (calcium silicate-based sealer OR calcium silicate-based root canal sealer) AND (root canal OR endodontics OR root canal treatment) OR (root canal filling OR root canal obturation). The references list of the included studies and previously published reviews were searched. Laboratory and clinical studies investigating at least one of the CSBS’ properties/outcome were included in the review. The studies performed in training simulated resin teeth or animal teeth were excluded.

### 1.2. Terminology

Rheological properties of calcium silicate-based materials such as ProRoot^®^ Mineral trioxide aggregate (MTA) (Denstply Sirona, Ballaigues, Switzerland) or Biodentine (Septodont, Saint-Maur-des-Fossés, France) were not appropriate to be used as a root canal sealer in association with gutta-percha for obturation. Therefore, in the past 10 years, specific root canal sealer formulations intended for this purpose were developed. These sealers are usually called “bioceramics” by most manufacturers for marketing purpose. This term is not accurate enough [6]. Indeed, chemically, bioceramics represent a large family of biomaterials in terms of composition, and further involve a sintering step in their implementation [23]. Therefore, this new family of root canal sealers should rather be identified as “calcium silicate-based sealers” (CSBS) or “hydraulic calcium silicate-based sealers”, due to their hydrophilic nature, chemical composition, and setting reaction [24]. CSBS are usually formulated from synthetic calcium silicate or from Portland/MTA. It is of prime importance to highlight that CSBS’ properties can strongly vary depending on the additives included in each formulation [25], and potentially influence their indications and clinical application.

## 2. Review

### 2.1. Physico-Chemical Properties 

#### 2.1.1. Setting Reaction and Setting Time

**Figure 1:**
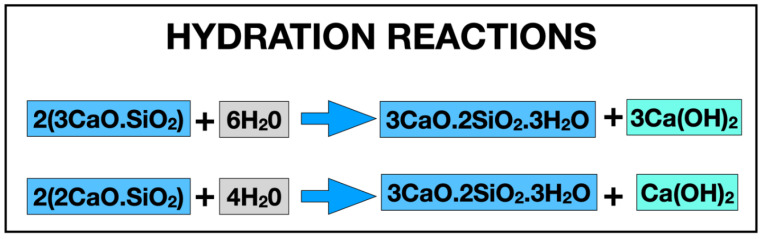
Setting reaction of CSBS consisting of two hydration reactions.

Unlike conventional sealers, CSBS are hydraulic and need water to trigger the setting process (Figure 1). In the presence of water, calcium silicates form a calcium silicate hydrate gel (CSH, CaO·SiO·H_2_O), which leads to calcium hydroxide (CaOH_2_) formation [26], as shown in Figure 1. Ion exchanges, predominantly silicon (Si^4+^) from CSH, and calcium (Ca^2+^) and hydroxyl (OH^−^) ions from calcium hydroxide dissociation, contribute to CSBS’ biological properties [7,8,10,12]. These ions provide different effects; Si^4+^ and Ca^2+^ promote biomineralization, while OH^−^ ions increase pH environment and provide antimicrobial properties. Finally, in the presence of phosphate, microscopic investigations showed that CSBS formed an interfacial layer at the dentin wall known as the “mineral infiltration zone” due to calcium phosphate formation inducing apatite precursors and hydroxyapatite precipitation on the surface of the material [24,27,28].

Setting time is evaluated by analyzing created indentations on a material sample’s surface; when indentations cease to be visible, setting time can be recorded [29,30]. CSBS overall reported a shorter setting time compared to conventional formulations such as AH Plus^®^ (Dentsply Sirona, York, PA, USA) [3,31]. However, prolonged setting times were also highlighted [32], depending not only on formulation, but also on root canal moisture, as it has been noted that when the root canal is dry, setting time tends to increase [18]. This explains why setting times vary between clinical trials and laboratory studies, and small amounts of fluids in contact with sealers may affect the latter [33]. For instance, it has been demonstrated in vitro that BioRoot™ RCS (Septodont, Saint-Maur-des-Fossés, France) had a setting time inferior to 6 h, while MTA Fillapex^®^ (Angelus, Londrina, Brazil) did not completely set within one week [34]. This lack of setting was also reported by another study [35] also investigating BioRoot™ RCS that indicated an influence of contact media (culture media) on the observed setting times. By contrast, when simulating different conditions (with an increased fluid intake), the setting time for both EndoSequence^®^ BC Sealer™ (Brasseler USA, Savannah, GA, USA) and MTA Fillapex^®^ was inferior to 3 h, which is much shorter than epoxy or zinc oxide–eugenol-based sealers [3]. Another study comparing EndoSequence^®^ BC Sealer™ and EndoSequence^®^ BC Sealer™ HiFlow (Brasseler USA, Savannah, GA), reported comparable initial setting times of 4 h for both formulations [36]. Although variable, these values remain generally lower than those of conventional sealers (zinc oxide–eugenol and resin-based). Finally, it was shown that applying heat during root canal filling resulted in an extended setting time for premixed CSBS such as HiFlow^®^ and Endosequence^®^ BC Sealer™, while the setting process was faster for BioRoot™ RCS, highlighting again the influence of the formulation on sealer properties [37].

#### 2.1.2. Flowability

Unlike the first calcium silicate-based materials, with inappropriate flowability/consistency for root canal filling [38], CSBS flowability should allow good sealer distribution into the ramifications/irregularities of the root canal space. The flow values are studied by placing a sample of mixed material between two glass plates with the application of a mass on top. At the end of the assay, the sample diameter is determined and used to assess the material flow capacity and must be superior or at least 17 mm [29,30] (ANSI/ADA, 2000; ISO 6876, 2012). Among available studies, it has been demonstrated that MTA Fillapex^®^, EndoSequence^®^ BC Sealer™, and Endoseal MTA^®^ (Maruchi, Wonju, Korea) [3,27,35,39,40] met the minimum expected values, and the highest values for MTA Fillapex^®^ were generally reported. However, while BioRoot™ RCS was characterized by results slightly below the minimum standard (16 mm) [13], it was also characterized as meeting the standard requirements with values above 21 mm [35], but decreasing with heat application [41]. HiFlow^®^ formulation exhibited the highest flow as compared to EndoSequence^®^ BC Sealer™, although it decreased with heat application [36]. Overall, based on the available literature, CSBS flowability should be considered as overall comparable to the conventional sealers, especially epoxy resin-based sealers such as AH Plus^®^.

#### 2.1.3. Wettability

Root canal sealers should have a good wetting ability and adhesion to dentinal walls [42]. Wettability reflects the spreading ability and the capability of sealers to penetrate into both the main and lateral canals, as well as into the dentinal tubules [43]. Since CSBS are hydrophilic, this might induce a good spreading ability on wet root canal walls [4]. This was confirmed by a recent study showed the best wetting ability and adhesion for EndoSequence^®^ BC Sealer™ and EndoSeal MTA^®^ compared to AH Plus^®^ [42].

#### 2.1.4. Film Thickness

Film thickness of tested material is determined under stress by placing the sample of the sealer between two glass slides and a load application. According to ISO6876/2012 and ANSI/ADA no 57, film thickness must not exceed 50 µm for sealers, as an end result of the test conditions [29,30]. This property is respected by various formulations such as EndoSequence^®^ BC Sealer™ HiFlow^®^, Endoseal MTA^®^, and MTA Fillapex^®^ [3,35,36], presenting overall higher values compared to AH Plus^®^. Moreover, BioRoot™ RCS exhibited the highest values of film thickness [35], and other studies described this property as slightly above the standard values [13,41]. Here too, film thickness values were reported to be increased by heat application for BioRoot™ RCS, EndoSequence^®^ BC Sealer™, and EndoSequence^®^ BC Sealer™ HiFlow^®^ [36,41]. Moreover, it can be considered that this characteristic for CSBS should be put in perspective with their better dimensional stability and their use with sealer-based obturation techniques such as cold hydraulic condensation (CHC).

#### 2.1.5. Dimensional Stability

CSBS dimensional stability is overall better than the one of conventional sealers, especially zinc oxide–eugenol-based sealers, which tend to shrink upon setting, especially if sealer film thickness increases [44,45,46]. It should be mentioned that this parameter is no longer present in the latest ISO standard. As initially demonstrated for MTA-based formulations, CSBS may present a slight hygroscopic expansion up to 0.2%, but this was not highlighted for all formulations [44].

**Figure 2:**
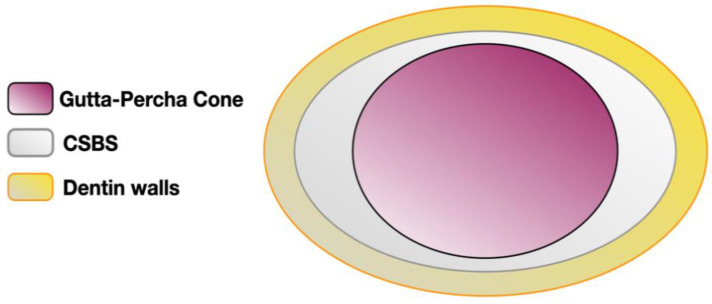
Updated single-cone technique with CSBS (sealer-based obturation) considering their enhanced dimensional stability.

Lee et al. (2017) compared dimensional stability between AH Plus^®^, AD Seal^®^ (Meta Biomed, Cheongju, Korea), and Radic-Sealer ^®^ (Seoul, Korea) and the CSBS formulation Endoseal MTA^®^. It was shown that AH Plus^®^ and Endoseal MTA^®^ revealed the least dimensional changes, especially for Endoseal MTA^®^, which remained lower than AH Plus^®^ 30 days later. The other two resin-based formulations had higher values than recommended [39]. In another study, no significant difference in volumetric change between AH Plus^®^ and TotalFill BC sealer was reported [27]. On the other hand, MTA Fillapex^®^ showed a slight shrinkage upon setting (which might have been due to the presence of resin in this formulation), while EndoSequence^®^ BC Sealer™ demonstrated an expansion, but inferior to 0.1% [3]. The expansion of EndoSequence^®^ BC Sealer™ might be influenced by direct contact of CSBS with enzymes [47]. By contrast, using micro-CT, a higher volumetric loss also was reported [32], but to a lesser extent with the use of PBS [26]. The better dimensional stability of CSBS is often highlighted as the main reason for allowing their use with cold hydraulic condensation, especially the single-cone (SC) technique (Figure 2). This aspect must also take into account the solubility of CSBS.

#### 2.1.6. Solubility of CSBS

Overall, CSBS solubility indicated higher values than those of conventional sealers without necessarily respecting the specifications of the standards (less than 3%) [29,30]. Systematically, studies reported that CSBS present higher solubility compared to epoxy resin-based sealers [3,26,27,32,34,35,36,47,48]. However, while some studies reported values of solubility with respect to ISO 6876/2012 and ANSI/ADA recommendations, others did not. Indeed, although the standard recommends using water, solubility values may strongly differ depending on experimental conditions such as setting conditions and contact liquid (water, PBS, culture media); for example, solubility reported for BioRoot™ RCS and MTA Fillapex^®^ fulfilled the standard recommendations (inferior to 3%), and the use of PBS lowered BioRoot™ RCS solubility [34]. This was also the case for MTA Fillapex^®^ and EndoSequence^®^ BC Sealer™ in the study of Zhou et al. (2013), which used a modified sample setting method and fulfilled the weight-loss requirements [3]. Another study indicated low solubility rates for EndoSequence^®^ BC Sealer™ and EndoSequence^®^ BC Sealer™ HiFlow formulations [36]. Moreover, solubility of EndoSequence^®^ BC Sealer™ was higher when in contact with biological fluids such as the Esterase enzyme as compared to PBS but remained in compliance with the ISO standard requirement in both conditions [47]. On the other hand, other studies have reported values much higher than the standard requirements (frequently above 10%), also using classical or various assay conditions, and concerned the previously mentioned CSBS formulations [26,27,32,35,48].

Investigation of CSBS’ solubility is a major matter of debate. Indeed, higher solubility of CSBS might lead to jeopardize their long-term sealing ability [5]. However, microscopic analysis has demonstrated mineral deposition and an infiltration zone into the dentin [26], which might call into question the above concern. Indeed, it must be pointed out that CSBS’ biological properties can be explained by their solubility and related release of ions [49], which leads to specific interaction between CSBS and the dentin walls (mineral infiltration zone). Furthermore, solubility may be overestimated due to the chemical class of CSBS, which could explain the discrepancies sometimes found between the high solubility values and the relatively lower ones concerning dimensional variations [27,32]. These contradictory results might be explained by the bias in the solubility of CSBS due to their hydrophilic nature. Moreover, since fluid environments (use of culture media) might strongly influence solubility results [35], it can be hypothesized that in vivo application of endodontic sealer should be relatively different with notably limited contact with aqueous fluids compared to in vitro test conditions.

#### 2.1.7. Adhesion–Interaction with Dentin Walls

CSBS adhesion and interaction with dentin walls were investigated by push-out test, filtration assays, or microscopy analysis. As mentioned previously, CSBS form a specific interfacial layer at the dentin walls known as the mineral infiltration zone [49]. The sealer’s hydration products alter the collagen of the interfacial dentin due to their alkaline effects [50]. This alteration leads to the formation of a porous structure promoting the diffusion of high concentrations of Ca^2+^, OH^−^, and CO_3_^2−^ ions, favoring mineralization in this area [18]. This chemical and micromechanical interaction (tag-like structures) represents the main reason for assessment of the adhesion between CSBS and dentin [49,51].

Laboratory studies found higher push-out bond strength (POBS) values for AH Plus^®^ when compared to MTA Fillapex^®^, TotalFill^®^ BC Sealer™, and BioRoot^TM^ RCS [52,53]. On the other hand, Tuncel et al. (2015) compared the POBS of AH Plus^®^ to iRoot SP^®^ (IBC, Burnaby, BC, Canada), and found that iRoot SP^®^ had significantly better results [54]. CSBS and conventional sealers showed variable results regarding bond strength and adhesion to the dentin walls; however, only one study showed no difference between CSBS and resin-based sealers [55]. Some variations have also been demonstrated between different CSBS formulations and depending on the root canal filling technique used; Delong et al. (2015) demonstrated that the lowest adhesion was found with MTA Plus^®^ (Prevest, Jammu, India) when warm obturation techniques were used. However, BC Sealer^®^ had higher bond-strength values than MTA Plus^®^ when both were used with the SC technique [56].

#### 2.1.8. Adhesion between the Gutta-Percha and the Sealer

CSBS are hydrophilic materials and the surface of gutta-percha cones is hydrophobic, which is why this interface remains questionable regarding potential micro-organism leakage [22]. Some manufacturers have proposed different strategies to enhance the adhesion between CSBS and gutta-percha. The use of specific pre-impregnated gutta-percha cones with “bioceramic” nanoparticles has been suggested with premixed formulations, while Septodont claimed the inclusion of an organic polymer (povidone) in their BioRoot™ RCS formulation. The only available study showed that the interface between these specific gutta-percha cones and the corresponding CSBS was not satisfactory [57]. Moreover, the contact between gutta-percha and sodium hypochlorite for disinfection before any obturation technique has been shown to degrade the gutta-percha cones [58]. This led us to wonder if specific coated gutta-percha cones may lose the claimed benefit when immersed in sodium hypochlorite. To our knowledge, there is no available scientific evidence supporting the use of specific pre-impregnated gutta-percha cones. Likewise, the effect of the povidone included in BioRoot™ RCS has not been investigated yet.

#### 2.1.9. Microhardness

Microhardness reflects the resistance of materials to deformation under a specific load. This property is not a part of the ISO/ADA requirements, and so it has been rarely investigated. Microhardness can be used as an indirect measurement of material setting [59]. The Vickers hardness test is used to assess the microhardness of sealers. Microhardness may impact CSBS removal when a non-surgical retreatment is indicated [22,59].

#### 2.1.10. Radiopacity

The ISO 6876 standard establishes 3 mm of aluminum (Al) as the minimum radiopacity for 1 mm root canal sealer sample thickness, as is the case of ANSI/ADA specification No. 57 [29,30]. Two main radio-opacifiers are generally included in CSBS formulations: Portland/MTA based-formulations most often contain bismuth oxide [60,61], whereas other CSBS generally include zirconium oxide in their formulations [38]. Overall, the standard specifications are respected in all CSBS formulations [62]. Different formulations of CSBS demonstrated higher radiopacity compared to the ISO standards. This was demonstrated for BioRoot™ RCS [13], EndoSequence^®^ BC Sealer™, EndosealMTA^®^, and MTA Fillapex^®^ [39]. TotalFill^®^ BC Sealer HiFlow™ might exhibit an additional radiopacity of 20% compared to standard TotalFill^®^ BC Sealer™ according to the manufacturer’s instructions (FKG Dentaire catalogue, La Chaux-De-Fonds, Switzerland).

### 2.2. Biological Properties

As previously presented, CSBS’ biological properties rely on a hydration reaction leading to CSH and calcium hydroxide formation. Indeed, hydration byproducts, OH^−^, Ca^+2^, and Si^+4^ ions are involved in modulating environment alkalization and cell metabolism, especially cell differentiation and tissue mineralization [63,64,65]. As a biomaterial, CSBS formulations must notably be non-genotoxic and non-cytotoxic, while also exhibiting antimicrobial properties and inducing appropriate host response in their specific use. These capacities, which rely on biocompatibility, are, among others, framed and evaluated through the ISO standard series 10993 [66]. Moreover, it is important to highlight that these studied properties, mostly in vitro, vary according to the protocols used. Indeed, biomaterial state (freshly mixed/set), type of contact (direct/extracts and associated dilutions), and targeted organisms chosen (cell lines/primary cell culture, planktonic bacterial strains/organized biofilms) will more or less accurately reflect the clinical use.

#### 2.2.1. Genotoxicity and Cytotoxicity

Genotoxicity is assessed using various protocols to study DNA breaks or nucleus division anomalies. In a study using a γ-H2AX foci assay, no difference in genotoxicity was highlighted between unset formulations of CSBS (BioRoot™ RCS, iRoot SP^®^, MTA Fillapex^®^) in comparison to conventional sealers (epoxy- and methacrylate-based), except a slight increase for iRoot SP^®^, while BioRoot™ RCS was revealed to be less genotoxic on periodontal ligament (PDL) cells [67]. However, when compared to a zinc oxide–eugenol formulation (Tubliseal), iRoot SP^®^ and EndoSequence^®^ BC Sealer ™ were shown to be the least genotoxic using a comet assay (DNA breaks) on L929 murine fibroblasts [68]. Furthermore, when human gingival fibroblast cultures were submitted to unset EndoSequence^®^ BC Sealer™, it led to a reduced genotoxicity potential as compared to AH Plus using a micronucleus assay [69]. Finally, set formulations of MTA Fillapex^®^ and AH Plus^®^, although depending on the concentration and the incubation time used, were shown to be more genotoxic by micronucleus assay on V79 fibroblasts as compared to classical MTA formulation [70].

In parallel, cytotoxicity was studied on PDL cells using unset biomaterial samples, and demonstrated a reduced effect of BioRoot™ RCS, iRootSP^®^, and MTA Fillapex^®^ as compared to other resin-based sealers such as AH Plus^®^. However, MTA Fillapex^®^ was revealed to be three times more cytotoxic than BioRoot™ RCS [67]. In another study, evaluating both freshly mixed and set sealer sample on human PDL cells, it was shown that BioRoot RCS was the least cytotoxic in both set and freshly mixed conditions, even allowing cell proliferation [71]. By contrast AH Plus^®^ was revealed to be cytotoxic in a freshly mixed condition, but not after setting, while MTA Fillapex and Pulp Canal Sealer (PCS) were characterized as cytotoxic in both fresh and set states [71]. Close results were obtained while comparing AH Plus MTA Fillapex^®^ and EndoSequence^®^ BC Sealer™ on gingival fibroblasts, indicating higher cell viabilities for EndoSequence^®^ BC Sealer™ in fresh/set conditions [72]. Conversely, AH Plus^®^ was more cytotoxic when freshly mixed, while MTA Fillapex^®^ was reported to be cytotoxic in both conditions [72]. Using set biomaterial samples, it was demonstrated on L929 murine fibroblasts by MTT assay that the zinc oxide–eugenol formulation was the more cytotoxic as compared to EndoSequence^®^ BC Sealer™ and iRoot SP^®^ [68]. Using direct contact with set biomaterial on isolated PDL cells, a much greater number of present cells for BioRoot™ RCS were demonstrated compared to a zinc oxide–eugenol (PCS) [12]. This has also been demonstrated on cell proliferation using sealer extracts, leading to a greater decrease with the use of PCS [12]. These results were confirmed in another study that used sealer extract on human PDL fibroblasts, and which demonstrated an increase of cell proliferation with the use of BioRoot™ RCS extracts as compared to PCS [73]. Moreover, a much lower CSBS cytotoxicity was also highlighted using an adenosine triphosphate luminescence assay on a murine osteoblast precursor cell line [74]. Indeed, AH Plus^®^ was revealed to be cytotoxic at concentrations a hundred times lower than EndoSequence^®^ BC Sealer™ and ProRoot ES (Dentsply Tulsa Dental Specialties, Tulsa, OK, USA) [74]. Cytotoxicity was also investigated in human PDL stem cells (PDLSCs) in two works by Collado-Gonzalez et al. that evaluated set biomaterial sample effects and indicated an overall cytotoxicity of MTA Fillapex^®^, Endoseal MTA^®^, and AH Plus^®^, while BioRoot™ RCS was characterized as highly biocompatible [7,75]. Similar findings have been reported in human PDLSCs by Rodríguez-Lozano et al., who concluded that TotalFill^®^ BC Sealer™ induced a lower cytotoxicity as compared to MTA Fillapex^®^ and AH Plus^®^ [76]. Finally, it was recently also demonstrated using sealer eluates from set biomaterials on PDLSCs that EndoSequence^®^ BC Sealer™ and EndoSequence^®^ BC Sealer™ HiFlow formulations were not cytotoxic, conversely to AH Plus^®^ [77].

#### 2.2.2. Antimicrobial Activity

CSBS’ antimicrobial activity is mostly linked to their ability to increase pH, as presented before, consecutive to hydroxyl ion releasing. Indeed, a pH increase was highlighted by many studies, in comparison to conventional sealer formulations [3,13,14,40,78,79]. Unlike the latter, CSBS induced an alkalization lasting in time, although this property was sometimes reported as reduced in the case of MTA Fillapex^®^. Evaluation of CSBS’ antimicrobial activity was also widely studied, using various protocols, micro-organism strains, and types of contact/micro-organism organization. Indeed, using set material sample for a direct-contact test on planktonic micro-organisms and a biofilm model on dentin, it was shown that TotalFill BC Sealer^®^ was more efficient against both *E. faecalis* and *C. albicans* [80]. In comparison with many other formulations, a fast and significant effectiveness of iRoot SP^®^ was shown just after mixing against *E. faecalis,* even after 3 days, conversely to AH Plus^®^ using a direct-contact test [81]. Regarding the antibacterial effect of CSBS, Candeiro et al. (2016) found a similar antibacterial effect of EndoSequence^®^ BC Sealer™ and AH Plus^®^ against *E. faecalis* using a direct-contact test up to 7 days [69]. Assessment against multiple bacterial strains in both a planktonic state and in simulated mono-specie biofilms, it was reported that TotalFill BC Sealer^®^ and AH Plus^®^ possessed antibacterial activity [82]. However, while AH Plus^®^ presented high antibacterial activity against all planktonic and biofilm bacteria strains during the first day, this property was drastically reduced for longer times. TotalFill BC Sealer^®^ use showed an antibacterial effect on planktonic strains up to 7 days, while its effect was lower on mono-specie biofilms, especially against *S. aureus* and *E. faecalis* [82]. Using an 8-week-old biofilm of *E. faecalis* in an infected root model, Bukhari and Karabucak demonstrated a superior antibacterial effect of EndoSequence^®^ BC Sealer™ after 1 day and up to 2 weeks, in comparison to AH Plus^®^ [83]. Antibacterial property was also studied depending on final irrigant use by an agar diffusion test and an intratubular infection model for BioRoot™ RCS, MTA Fillapex^®^, and AH Plus^®^ against *E. faecalis*. It was concluded that the formulations exhibited higher antimicrobial effects after EDTA use as compared to PBS, and that BioRoot™ RCS exhibited the highest activity [84].

Overall, CSBS presented similar or even higher antimicrobial properties than conventional sealers. However, a lack of standardization for assessment of antimicrobial properties has been highlighted [85]. Moreover, it must be pointed out that the clinician should rely on the root canal disinfection/cleaning procedure instead of the antibacterial properties of endodontic sealers.

#### 2.2.3. Bioactivity

Although a biomaterial can be characterized as biocompatible, its bioactivity qualification implies an ability to stimulate metabolic/cellular-specific events, leading to tissue healing, whether through regenerative step induction, inflammation control, or both. In the case of endodontic sealers, events such mesenchymal stem cell migration, growth factor secretion, and cell differentiation are implicated in periapical healing, just as the modulation of pro-inflammatory factor cell secretion/expression or immune cells recruitment are related to periapical inflammation resolution.

Jung et al. (2018) showed in two studies that in comparison to PCS, AH Plus^®^, and MTA Fillapex, only the BioRoot™ RCS had a positive influence on cell metabolism of both PDL cells and osteoblasts [71,86]. Furthermore, human PDLSC activity and migration were evaluated using a scratch wound healing assay and adhesion to collagen type I with set sealer eluates of TotalFill BC Sealer^®^, MTA Fillapex^®^, and AH Plus^®^ [76]. Results indicated the most-favorable responses with the use of TotalFill BC Sealer^®^, while the use of MTA Fillapex^®^ resulted in the least-favorable responses, even compared to AH Plus^®^ [76]. All of these previously mentioned cell populations are essential in periapical tissue regeneration, and alteration of their metabolism/activity may impact this latter. Evaluating PDL lipopolysaccharides (LPS)-stimulated fibroblast implication in both regeneration and inflammation events, it was demonstrated that BioRoot™ RCS, conversely to PCS, did not alter PDL stem cell migration while controlling immune cell (THP-1 model) migration and activation. Furthermore, this study highlighted that BioRoot™ RCS induced PDL fibroblast growth factor (TGF-β1) secretion and reduced pro-inflammatory cytokine (IL-6) secretion by ELISA [73]. It has also been shown that the use of BioRoot™ RCS did not alter the cell mesenchymal character and migration ability of human PDLSCs [7]. Moreover, PDL cell angiogenic/osteogenic growth factor secretions (VEGF, FGF, BMP-1) were shown to be increased by the use of BioRoot™ RCS extracts [12]. In addition to their secretion, it has also been shown that the expression of osteogenic factor by murine osteoblast precursor cell line was increased by EndoSequence^®^ BC Sealer™ and ProRoot ES, using fluorescence and RT-PCR (DMP-1, ALP), while the use of AH Plus^®^ impaired this osteogenic potential [74]. However, using diluted material extracts of EndoSequence^®^ BC Sealer ™, MTA Fillapex^®^ and AH Plus^®^ both increased the cell osteogenic potential of an osteoblast cell line after an LPS-induced inflammation state [87]. Moreover, in addition to an osteogenic potential, it has also been demonstrated by qPCR that the EndoSequence^®^ BC Sealer™ and HiFlow formulations were able to stimulate human PDLSC mineralization and cementogenic marker expressions (ALP, CEMP, RUNX2, and CAP), while AH Plus^®^ did not [77]. Concerning the inflammation process, the effect of iRoot^®^ SP use was studied on macrophage viability, cytokine expression, and macrophage polarization [88,89]. Indeed, the inflammatory reaction is a complex process, and while often considered to be deleterious, is necessary for the implementation of the regeneration steps, and macrophage polarization plays an important role. Indeed, the macrophage M1 phenotype is recognized as pro-inflammatory, while shifting to the M2 phenotype acts as anti-inflammatory [90]. Zhu et al. demonstrated that iRoot^®^ SP was not cytotoxic for a model of macrophage (RAW 264.7) and induced both pro- and anti-inflammatory cytokine expressions (IL-1b, TNF-a, IL-10, IL-12p40). Moreover, use of this CSBS formulation induced an increase of M1 and M2 macrophage marker expression and reduced the balance of M1/M2 macrophage phenotypes, indicating that this sealer could promote healing processes [89]. Close results were obtained by Yuan et al., who studied iRoot^®^ SP’s effects on the same events after an LPS-induced inflammatory state simulation. This work also found a potential effect of iRoot^®^ SP on mRNA inflammation factor expressions and M1/M2 macrophage phenotype balance [88].

Taken together, the whole of these in vitro studies, clearly demonstrated that CSBS, presented promising biological properties, when compared to conventional sealers. It may hypothesize that, in addition to an adequate endodontic clinical protocol, CSBS could promote the healing process in case of apical periodontitis due to their enhanced biocompatibility and certain bioactivity. However, it must be pointed out that additive in formulations can alter these properties. Indeed, more inconsistent results in the literature were obtained with MTA Fillapex^®^ formulation. This is often explained by the presence of resinous compounds of the salicylic type in their formulations and substance leaching [72,91,92], just as a silicate hydration reaction alteration and reduced or absent calcium hydroxide formation [25].

### 2.3. Obturation Quality

The main objective of obturation is to prevent leakage and reinfection of the root canal system [93]; microleakage can occur due to gaps or voids occurrence [94,95]. While the postoperative radiograph helps in assessing the obturation quality in a clinical approach, many laboratory methods can value the root canal filling quality in vitro: dye penetration, dye diffusion, bacterial and endotoxin infiltration, electrochemical, microscopy, or 3D evaluation [62]. Voids are often investigated because they represent some spaces where residual bacteria might re-grow and release their byproducts, thus jeopardizing the long-term success of the root canal treatment [96,97].

A study evaluating apical sealing ability using apical linear dye penetration and comparing AH Plus^®^, Endosequence BC^®^ Sealer™, and MTA Fillapex ^®^ showed the lowest apical leakage value for the SC technique used with the EndoSequence BC^®^ Sealer™ [98]. As already shown in the literature, results for the dye techniques remain contradictory, inducing a wide variability. An important consideration in relation to dye penetration studies is that air trapped in voids within the root canal obturation material might interfere with fluid movement [62,99].

One study evaluated the microleakage of different types of sealer, demonstrating that the Endosequence BC^®^ Sealer™ group showed the least dye leakage, while the highest leakage was observed in zinc oxide–eugenol-based sealer [100].

**Figure 3:**
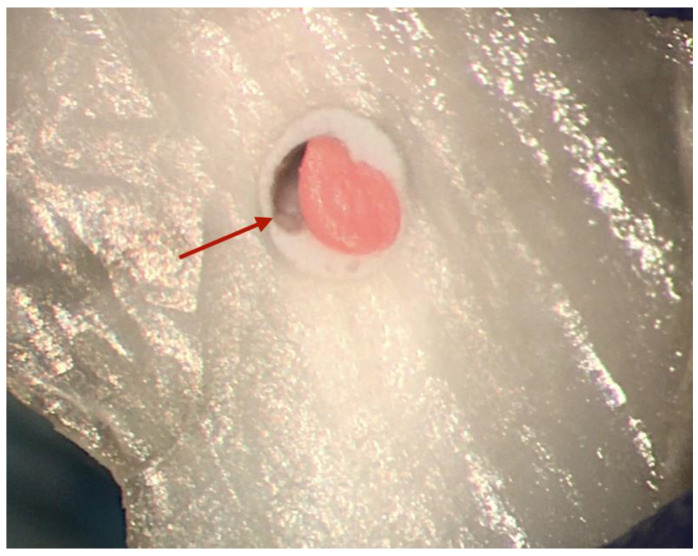
Large void following root canal obturation with single cone technique.

Nevertheless, many factors may influence voids’ proportion, including the root canal filling technique (Figure 3), film thickness, flowability, and wettability.

Void incidence has been reported to be greater within oval root canals, especially when this space was filled with CHC and especially when using the SC technique or cold lateral compaction [101,102]. Another study assessed the filling quality of five obturation techniques in oval-shaped root canals by using an optical numeric microscope, SEM, and energy-dispersive X-rays (EDX) [103]. This study investigated the proportions of gutta-percha-filled areas, sealer-filled areas, void areas, and the sealer/gutta tags into dentinal tubules. Obturation quality was overall better when using a warm gutta-percha obturation technique compared to the use of the SC technique, regardless of the type of sealer. A recent study based on confocal microscopic evaluation showed that the use of warm vertical compaction enhanced the penetration of CSBS into the dentinal tubules in comparison with the SC technique [104]. The inherent limitations of the SC technique even using CSBS was demonstrated in a micro-CT study [105].

Micro-CT has been suggested to be the most reliable technique to investigate the filling quality differentiating gutta-percha, sealer, and voids. This technique allows the evaluation of void/porosity incidence (apical, middle, or coronal thirds), and the identification of their type (internal, external, or combined) [106,107]. A study assessed the remaining voids after obturation between Endosequence^®^ BC Sealer™ and AH Plus^®^ using the SC technique. EndoSequence^®^ BC Sealer™ showed a lower ratio of voids compared to AH Plus^®^ in the apical third, but it was highlighted by the authors that this difference was likely due to root canal anatomy variations [108]. A recent study showed that the proportion of open and closed porosity can change over time [107]. Initially, significantly greater open and total porosity were found for MTA Fillapex^®^ than for AH Plus^®^. After 6 months, the percentage of open and total porosity increased in BioRoot™ RCS and MTA Fillapex^®^, and decreased in AH Plus^®^ and Endosequence^®^ BC Sealer™. These findings were explained by the greater solubility of BioRoot™ RCS and MTA Fillapex^®^ compared to AH Plus^®^. The better ability of EndoSequence^®^ BC Sealer™ to create apatite formation compared to BioRoot™ RCS might explain the reduction of porosity for EndoSequence^®^ BC Sealer™ 6 months after storage [107].

When compared to conventional sealers, CSBS have overall shown comparable results when evaluating void incidence using micro-CT [109]. However, void incidence should be always put in perspective with the root canal anatomy and the obturation technique used.

### 2.4. Retreatability

Non-surgical retreatment implies removal of root canal filling material in order to re-establish apical patency, then clean and fill the entire root canal system (AAE 2012). Therefore, retreatability is one of the requested properties of filling materials [110,111]. Currently there is no technique allowing complete removal of filling materials from a root canal system [111]. In addition, several factors may influence the retreatability, such as the filling technique implemented, and the type of sealer used with gutta-percha [110,111].

CSBS are known to be hard upon setting [112] and to create hydroxyapatite crystals upon their interface with dentin [113]. In addition to that, they are capable of penetrating into the dentinal tubule. These properties may render retreatment procedures difficult [114]. To study removal of filling materials, different methods have been used such as micro-computed tomography (micro-CT), cone-beam computed tomography (CBCT), radiography, tooth splitting and direct visualization by SEM, confocal microscopy, stereomicroscopy or digital cameras, and rendering the teeth transparent [110,114,115,116,117]. As it has already shown to be reliable for evaluation of the quality of the root canal filling, micro-CT is non-invasive and allows for the comparison of the remaining volume of the filling material to the initial volume. In addition to visualizing and measuring the remaining filling material, SEM and confocal microscopy can also be used to assess the degree of penetration of the sealer inside dentinal tubules, or to quantify the number of open tubules [114,116].

Ersev et al. (2012) compared the retreatability of four root canal sealers (Hybrid Root SEAL, EndoSequence^®^ BCSealer™, the Activ GP system, and AH Plus^®^) and found no significant differences between the different sealers, or between the techniques used [118]. As demonstrated in many investigations, no technique allowed the complete removal of the filling material. Simsek et al. (2014) compared the number of opened tubules using SEM after the removal of iRoot^®^ SP, AH Plus^®^, and MM Seal^®^ in straight premolars filled with the lateral compaction technique after the use of R-endo rotary instruments or ESI ultrasonic tips. Likewise, no group showed complete removal of the filling material, with greatest leftover in the apical third [116].

Kim et al. (2015) also did not find any significant differences between Endosequence^®^ BC Sealer™ and AH Plus^®^ when comparing the amount of residual material using SEM analysis [114]. According to Uzunoglu et al. (2015), more remaining filling material was observed following the SC technique with iRoot^®^ SP compared to SC with AH-26^®^ or lateral compaction with AH-26^®^ (DeTrey, Dentsply Maillefer, USA), when assessed with SEM [110]. In addition, Suk et al. (2017) did not find any significant differences in the removability of EndoSequence^®^ BC Sealer™ and AH Plus^®^. In this study, MTA Fillapex^®^ was found to be the easiest to remove [117].

Hess et al. (2011) noted better removability of AH Plus^®^ compared to Endosequence^®^ BC Sealer™ in canals of less than 20 degrees of curvature [119]. More remnants of this CSBS were found in the apical third upon SEM analysis, and patency was not re-established in 20% of samples with BC Sealer and master cone to the WL, or in 70% of samples with BC Sealer and master cone short of the WL. Agrafioti et al. (2015) compared the retreatability of Total Fill^®^ BC Sealer™, MTA Fillapex^®^, and AH Plus^®^ in straight canals [113]. Authors have demonstrated that WL and apical patency were re-established in 100% of cases, when the gutta-percha cones were placed at WL. Oltra et al. (2017) compared the retreatability of BC Sealer and AH Plus^®^ using micro-CT imaging and found that the latter was associated with less residual filling materials, and that the use of chloroform may help BC Sealer removal [120]. On the other hand, Donnermeyer et al. (2018) found that AH Plus^®^ was associated with more remnants when compared to Bio Root™ RCS, MTA Fillapex^®^, and Endo CPM (Egeo, Buenos Aires, Argentina) [112].

Contradicting results between studies [112,120] could be related to the application of different methodologies, especially the length of adjustment of the gutta-percha cone and the dental sample anatomy. In the study conducted by Hess et al. (2011), gutta-percha cones were intentionally placed short of the apical foramen. It must be pointed out that this method represented the most realistic scenario of a non-surgical retreatment. This could clearly compromise retreatment outcome [119]. In other studies, gutta-percha cones were placed at full WL This different protocol could strongly influence the ability to re-establish the apical patency after removal of root canal filling material. Indeed, with the gutta-percha cone being introduced to the full working length, the apical patency could easily be re-established following easy removal of the latter. However, these situations did not correspond to the vast majority of retreatment indications. Indeed, it is well known that apical periodontitis is usually diagnosed in the case of poor quality and short obturation [121].

On the other hand, root canal anatomy, such as canal curvature and cross-section, may also impact retreatability. Hess et al. (2011) used mesial canals of mandibular molars, while in Agrafioti et al. (2015), straight canals from anterior teeth were evaluated [113,119].

In addition, the obturation technique used can influence the results. Manufacturers usually recommend CSBS with the SC technique, and some studies demonstrated that the use of these sealers with continuous wave condensation may decrease their bond strength [56]. This may explain the absence of differences between CSBS and resin-based sealers in the studies conducted by Agrafioti et al. (2015) and Kim et al, (2015) [113,114].

Contradictory results were also obtained regarding the retreatment time. Simsek et al. (2014) did not find a statistical difference in the time to reach WL when removing iRoot^®^ SP, MM Seal, and AH Plus^®^ [116]. Similar findings were obtained by Kim et al. (2015) when comparing time for removal of EndoSequence^®^ BC Sealer™ and AH Plus^®^ [114]. Uzunoglu et al. (2015) reported a faster retreatment when the filling material consisted of gutta-percha and MTA Fillapex^®^ compared to AH Plus^®^ and iRoot^®^ SP, which showed similar results [110]. Donnermeyer et al. (2018) found that the removal of CSBS (BioRoot™ RCS and Endo CPM) was faster than for AH Plus^®^ [112].

In conclusion, most ex vivo studies showed possible CSBS removal, and an ability to regain apical patency in the majority of cases. However, methodological bias could be observed in many studies, and further studies better simulating retreatment indications and conditions are needed.

## 3. A Proposal for Clinical Perspectives on CSBS with Cold Hydraulic Condensation

### 3.1. Root Canal Anatomy

CHC and cold lateral compaction are known to increase void occurrence compared to warm gutta-percha obturation techniques, especially in large and oval canals regardless of the type of sealer [103,105,122,123]. However, in case of narrow, long, and curved canals, the use of warm vertical compaction can be questionable, since penetration of the heat plugger at the appropriate level (4 mm short of the working length) can sometimes be impossible. Thus, the gutta-percha is not heated and melted in the apical third, and the obturation of this area behaves as a SC technique [124]. Using CHC with CSBS in these types of anatomy makes root canal obturation easier and faster while taking advantage of CSBS’ physico-chemical and biological properties.

### 3.2. Operative Accessibility

**Figure 4:**
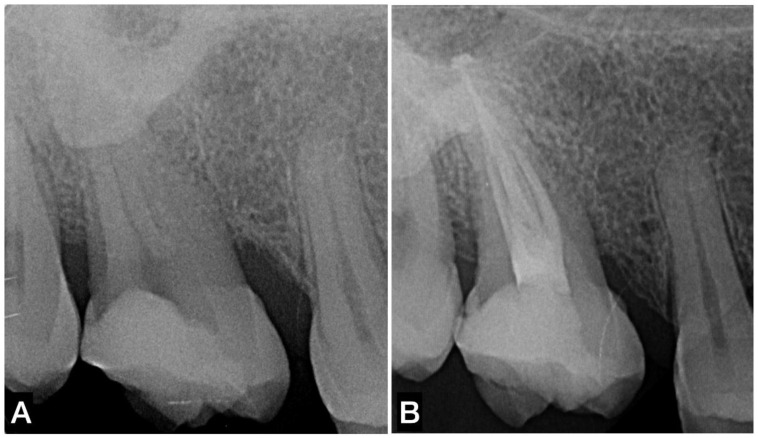
Example of indication of the single-cone technique with CSBS. (**A**) Preoperative periapical radiograph of a necrotic maxillary molar with long roots, sinus proximity, and patient’s limited mouth opening. (**B**) Postoperative periapical radiograph of root canal obturation using CSBS.

It is common sense to highlight that CHC and CSBS should make the obturation procedure easier and faster when dealing with a restricted access (limited mouth opening/posterior teeth) compared to the use of thermoplasticized gutta-percha obturation techniques. Indeed, by using CHC, the technical difficulties are limited to the intracanal sealer placement and the insertion of the gutta-percha cones (Figure 4).

### 3.3. Biological Aspects

As mentioned previously, their biological properties are the main advantages of CSBS over conventional sealers. A recent international survey showed that this has been claimed to be the most-frequent reason to justify their clinical use [22]. Based on the findings of in vitro studies, CSBS antibacterial activity and biomineralization ability might have the potential to stimulate and improve the periapical healing, and thus should be suitable in the case of apical periodontitis. Likewise, CSBS alkalization ability and calcium hydroxide formation might make them interesting to use in the case of external inflammatory root resorptions.

Finally, even if sealer extrusion in the periapical area is not suitable and should remain inadvertent, a sealer “puff” during obturation can be difficult to predict and control [125]. Taking into consideration better CSBS biological properties over the ones of conventional sealers highlighted in this narrative review, CSBS might be preferable to use in the following situations:

- Connection between the roots and the maxillary sinus, especially for immunocompromised patients for whom zinc oxide–eugenol-based and formaldehyde-based sealers are not recommended [22].
- Connection between the roots and inferior alveolar nerve: CSBS are more biocompatible, and their use with CHC avoids thermal nerve injuries.
- Middle or apical root canal perforations, consequences of a false canal: the use of CSBS with CHC allows the filling of the root canal and the perforation at the same time while also taking advantage of their biological properties.
- Patients with high risks of osteonecrosis connected to treatments such as radiotherapy or anti-resorptive drugs such as bisphosphonates, because it is suitable to reduce bone aggression factors in these situations.

However, it must be highlighted that regarding the biological aspects, a direct translation from the findings of in vitro studies to clinical outcome is not relevant. Indeed, the healing of the periapical area is not only related to the sealer’s choice, but involves numerous complex mechanisms, including the patient’s immune system [126]. 

## 4. Clinical Application of CSBS

### 4.1. Can CSBS Be Used with Any Type of Gutta-Percha?

Based on our review of the literature investigating the interface between gutta-percha and CSBS, there is no available evidence supporting the use of specific pre-impregnated gutta-percha cones with CSBS. However, a different interface quality between CSBS and the gutta-percha cone might be observed, depending on the type of gutta-percha and related chemical composition [22,57,127].

### 4.2. Do CSBS Usage Impact the Final Irrigation Protocol and the Root Canal Drying Technique?

Intracanal moisture negatively influences the setting process of conventional sealers and their adhesion to dentinal walls [128]. Unlike them, CSBS need water to initiate the hydration reaction that conditions their setting process, and also their biological properties [4]. According to the manufacturers, the dentinal tubules’ moisture initiates the setting of premixed formulations [4]. Therefore, intracanal dentin desiccation should be avoided, leading to gently dry the root canal before obturation [129]. This procedure is difficult to control, as it was shown in restorative dentistry in a wet-bonding procedure [130]. The use of intracanal micro-suction to empty the canal before the use of one sterile paper point could help preventing over-dehydration [129]. On this basis, a final rinse with ethanol is contra-indicated when using CSBS [22,129].

Finally, since the canal has to remain slightly wet, potential interactions between the final irrigant and CSBS should be taken into account. Indeed, several studies showed that most of the available irrigants (NaOCl, CHX, EDTA) may negatively affect CSBS [52,84,131]. So far, the clinical significance of such interactions remains unclear. However, it seems suitable to perform a final rinse with sterile water to flush out the last irrigant before root canal drying.

### 4.3. How to Reduce Voids Occurrence When Using CSBS with CHC?

As mentioned previously, the presence of open porosity occurring at the interface between the sealer and dentinal wall/gutta-percha may constitute a space for residual micro-organisms to regrow and leak toward the periapical area [107,132].

SC obturation induces a higher void ratio compared to warm obturation techniques, especially in oval or wide root canals [103]. However, as reported in the literature, all the filling techniques investigated are never “void-free” regardless of the type of sealer used [133,134]. When dealing with CHC, especially the SC technique, more emphasis is put on the sealer than the gutta-percha (sealer-based obturation concept). Although the intracanal sealer placement technique might impact void incidence, the latter is rarely specified in most publications. Many techniques can be used to place CSBS into the root canal system, depending on the formulation and the anatomy:

- Coating the master cone with CSBS followed by its slow insertion to the full working length. This technique might be insufficient when dealing with oval or wide canals. Accessory cones can also be used to complete the sealer distribution.
- Lentulo spiral usage at low speed (around 700–800 rpm) or flexible injection tip before master cone insertion.

Applying sonic/ultrasonic activation and other sealer activation/agitation procedures may also contribute to improve CSBS distribution in the root canal space [135], but the level of evidence on these points is still weak.

### 4.4. Can CSBS Be Used with Thermoplasticized Gutta-Percha Obturation Techniques?

As stated previously, the SC technique being associated with greater void incidence, using CSBS with thermoplasticized gutta-percha obturation could make sense, as this would combine the advantages of these techniques already used by many endodontic specialists with the improved properties of CSBS. However, this leads us to question the impact of heat on CSBS’ properties, which have been addressed in several studies showing different findings according to the formulations tested [25,37,136,137]. A temperature rise (especially above 100 °C) may lead to a change in CSBS’ physical properties, especially their flowability, setting time, and adhesion to dentin walls [104,136]. Based on the available knowledge, Endosequence^®^ BC Sealer™ HiFlow^®^ and EndoSequence^®^ BC Sealer™ formulations could be used with heat [104], but not all CSBS can. For instance, BioRoot™ RCS is contra-indicated with warm gutta-percha obturation [25,37]. Therefore, there is a need for additional studies to clarify the impact of heat on each CSBS formulation. These considerations should also take into account the real temperature delivered by the heater plugger, which has been reported to be much lower than the one displayed on the device screen [137]. Finally, conventional sealers have also been reported to be negatively impacted by heat application in laboratory studies [37], while they have been used widely for decades with thermoplasticized gutta-percha obturation techniques and with satisfactory clinical outcome. This points out the gap existing between the findings of in vitro studies and the complexity of parameters involved in the clinical outcome.

### 4.5. Does Use of CSBS Make Non-Surgical Retreatment More Difficult?

The literature showed that CSBS may be removed with difficulty in the case of retreatments [119]. No specific solvent is available for removing CSBS during retreatments, even if formic acid and chloroform may help the endodontist. As stated previously, studies assessing CSBS retreatability have shown that apical patency could be properly achieved when the obturation of the previous treatment reached the full working length [112,118,138,139]. Nevertheless, non-surgical retreatments are mainly indicated when the obturation is short. Good flowability of CSBS may result in CSBS penetration beyond the gutta-percha cone tip. The presence of CSBS only and its hardness may make apical patency much more challenging to achieve, especially in curved root canals [119] blocking the access to the apical third and resulting in possible procedural errors such as ledges. Furthermore, retreatments also aim to remove all previous materials and disinfect the root canal system before filling it again. Nevertheless, the complete removal of the obturation material remains impossible, and all the techniques shown in the literature were only able to partially remove CSBS from the root canal [114,117] as demonstrated with any filling material.

## 5. Conclusions

This narrative review aimed to discuss the properties of CSBS and their clinical implications, and to propose rational indications based on the current knowledge. This work may help practitioners in selecting the appropriate sealer and pave the way for reasoned CSBS usage. CSBS have shown good all-around performance when compared to conventional sealers, but significant differences could be observed between the different CSBS formulations. Their particularity remains in their interesting biological properties, which were proven to be better than those of conventional sealers. However, the clinical impact of CSBS solubility must be clarified in the future. Likewise, available CSBS formulations can present specificities that have to be considered by the practitioner for proper clinical usage. Finally, the usual clinical endodontic protocol has to be slightly revised to consider CSBS specific behavior.

## Data Availability

No new data were created or analyzed in this study. Data sharing is not applicable to this article.
